# Regulatory Effects of RNA–Protein Interactions Revealed by Reporter Assays of Bacteria Grown on Solid Media

**DOI:** 10.3390/bios15030175

**Published:** 2025-03-08

**Authors:** Guillermo Pérez-Ropero, Roswitha Dolcemascolo, Anna Pérez-Ràfols, Karl Andersson, U. Helena Danielson, Guillermo Rodrigo, Jos Buijs

**Affiliations:** 1 Ridgeview Instruments AB, 75237 Uppsala, Swedenjos@ridgeview.eu (J.B.); 2 Department of Chemistry—BMC, Uppsala University, 75123 Uppsala, Sweden; 3 Institute for Integrative Systems Biology (I2SysBio), Centro Superior de Investigaciones Científicas (CSIC)—University of Valencia, 46980 Paterna, Spain; 4 Department of Biotechnology, Polytechnic University of Valencia, 46022 Valencia, Spain; 5 Giotto Biotech SRL, 50019 Sesto Fiorentino, Italy; 6 Magnetic Resonance Center (CERM), Department of Chemistry Ugo Schiff, Consorzio Interuniversitario Risonanze Magnetiche di Metalloproteine (CIRMMP), University of Florence, 50019 Sesto Fiorentino, Italy; 7 Department of Immunology, Genetics, and Pathology, Uppsala University, 75185 Uppsala, Sweden; 8 Science for Life Laboratory, Drug Discovery & Development Platform, Uppsala University, 75123 Uppsala, Sweden

**Keywords:** real-time protein expression, RNA–protein interaction, RBP, Musashi-1, reporter assay, agar

## Abstract

Reporter systems are widely used to study biomolecular interactions and processes in vivo, representing one of the basic tools used to characterize synthetic regulatory circuits. Here, we developed a method that enables the monitoring of RNA–protein interactions through a reporter system in bacteria with high temporal resolution. For this, we used a Real-Time Protein Expression Assay (RT-PEA) technology for real-time monitoring of a fluorescent reporter protein, while having bacteria growing on solid media. Experimental results were analyzed by fitting a three-variable Gompertz growth model. To validate the method, the interactions between a set of RNA sequences and the RNA-binding protein (RBP) Musashi-1 (MSI1) were evaluated, as well as the allosteric modulation of the interaction by a small molecule (oleic acid). This new approach proved to be suitable to quantitatively characterize RNA–RBP interactions, thereby expanding the toolbox to study molecular interactions in living bacteria, including allosteric modulation, with special relevance for systems that are not suitable to be studied in liquid media.

## 1. Introduction

Proteins that interact with RNA are well-known mediators of a wide array of cellular functions, such as cell growth and differentiation [1,2,3]; these are also involved in the development of different diseases, from cancer to Alzheimer’s disease [4,5,6,7]. These interactions regulate mRNA translation by enhancing its stability or favoring degradation [8]. Therefore, they have been the subject of an intensive characterization effort [9] that has opened the door to the development of new therapies [10] and synthetic biology applications [11].

Several methods have been used to study RNA–protein interactions, including in vivo, in vitro, and in silico approaches [12]. To study the interactions with isolated RNA-binding proteins (RBPs), biophysical in vitro techniques, such as nuclear magnetic resonance (NMR) [13], electrophoretic mobility shift assay (EMSA) [14], fluorescence anisotropy [15], and surface plasmon resonance biosensors (SPR) [16,17], have been used. To study interactions in a biologically more relevant cellular environment, in vivo assays are required. The most common technique to discover and study interactions between proteins and RNA is the crosslinking and immunoprecipitation assay (CLIP), which allows the characterization of interactions in a native environment [18]. There are several modifications of the original technique [19,20,21], with the Kin-CLIP approach able to quantify the interaction kinetics [22]. Reporter assays are also used to characterize interactions between biomolecules, including RNA–protein interactions [23]. These assays are based on the transformation of cells with a reporter molecule, whose expression is tuned depending on the interaction between biomolecules. A standard setup would be to transform bacteria with a plasmid encoding a fluorescent protein whose expression is controlled by an interaction between two biomolecules [24]. Experiments are typically performed with bacteria in suspension and a fluorescence readout. However, the use of solid media, such as agar, is becoming more common due to better expression conditions [25,26,27] and the possibility of studying more complex environments, as biofilms, which are relevant to the characterization of new antibiotics [28].

LigandTracer technology is widely used for real-time cell-binding assays (RT-CBA) and typically used to measure the binding kinetics and calculate the affinity of fluorescent labeled proteins for receptors on living mammalian cells that are adhered or tethered to the surface of a Petri dish [29]. These measurements can be performed in environment-controlled cabinets, like heat chambers or incubators, to create relevant assay environments and allow to follow the accumulation of fluorescently labeled proteins for many hours. The fluorescent readout can also be used to monitor green fluorescent protein (GFP) accumulation in cells [30] and is thus potentially useful as a GFP-based reporter assay. Moreover, as the excitation of fluorescent molecules on a layer of cells is on the same side as detection of fluorescent emission, a LigandTracer assay is not affected by the optical opaqueness of the solid support, such as agar, on which cells are grown. So far, bacteria assays have been limited to interaction studies where bacteria were captured by an adsorbed antibody layer under conditions that minimize cell growth [31].

In this study, we developed a method that enables continuous monitoring of superfolder GFP (sfGFP) expression as a reporter gene with high temporal resolution. It works with sessile bacteria grown on agar. The solid support-based reporter gene assay was developed, optimized, and evaluated with a system that reports on the interaction between the RBP Musashi-1 (MSI1), and several RNA strands selected on previous in vitro binding studies [32]. MSI1 plays a key biological role as a regulator of stem cell differentiation in neural cells [33] and its regulation is relevant in several diseases ranging from cancer [34,35,36] to Alzheimer’s disease [37]. MSI1 is part of the RNA recognition motif (RRM) family, harboring two RNA-binding sites (RRM-1 and 2) [38] that display a typical αβ sandwich structure with a βαββαβ distribution [39]. Both RNA-binding sites interact with UAG motifs in RNA strands with an affinity ranging from 15 to 50 nM; the interaction is characterized by relative fast association and dissociation rates, and readily forms bivalent complexes [17]. MSI1-RNA interaction is inhibited by fatty acids (with a higher affinity for mono-unsaturated fatty acids) [40], and the binding to RNA is also regulated by protein–protein interactions in mammals [41]. Although MSI1 is a key regulator of processes in certain eukaryotic cells, it is not endogenously expressed in bacteria and therefore has no endogenous interaction partners. This was an advantage for the current study since it could then be based on engineered bacteria expressing MSI1 as a reporter system.

The employed regulatory system consists of two different plasmids. One contains the coding sequence of the folded domain of MSI1 (1-192 amino acids), expressed under the control of the PLlac promoter, which is induced by the presence of lactose or lactose analogues. The other plasmid contains the RNA sequence recognized by MSI1, fused to the sfGFP N-terminal coding region, which acts as the reporter. As a standard experimental setup, bacteria previously transformed with the regulatory system to be assayed were grown on agar while continuously monitoring the fluorescence of sfGFP with high temporal resolution. Different RNA-binding motifs (mutants) as well as the allosteric modulation of MSI1 by oleic acid were evaluated. Our results were compared to a previous biophysical characterization of MSI1-RNA interaction and a previous synthetic biology study employing bacteria in suspension [42].

## 2. Materials and Methods

*Strains*, *plasmids*, *reagents*. *E. coli* DH5α was used for cloning purposes following standard procedures. To express the regulatory circuit comprised by both plasmids for LigandTracer assays, *E. coli* BL21(DE3) cells (lacI+, T7pol+) were used. This strain was co-transformed by chemical transformation with two plasmids as described previously [42]. One plasmid contains the gene coding for the RNA-binding protein of interest, i.e., the truncated version of MSI1 (the first 192 amino acids with the two RRMs) and expression is controlled by the inducible promoter of PLlac. The other plasmid contains the reporter gene fused with RNA sequences that are recognized by MSI1 followed by the gene coding for sfGFP. Single colonies were inoculated onto 2 mL of Lysogeny Broth (LB) supplemented with antibiotics. Cell cultures were grown overnight at 37 °C with orbital shaking (220 rpm, InforsHT Minitron, Basel, Switzerland). Isopropyl β-D-1-thiogalactopyranoside (IPTG) was used at the concentration of 1 mM. Kanamycin (Sigma-Aldrich, Darmstadt, Germany) and chloramphenicol (Sigma Aldrich) were used as antibiotics at a concentration of 50 μg/mL and 34 μg/mL, respectively.

*Dish coating.* Untreated MultiDish 2 × 2 (1-04-202, Ridgeview Instruments, Uppsala, Sweden) was coated with LB agar. MultiDishes were chosen as support because it has four independent sections that enable monitoring and comparing four different experimental conditions per assay. Each section of the MultiDish was coated with 4 mL LB agar with the suitable reagent. The coated MultiDish was incubated at room temperature until the agar was solidified.

*Dish seeding*. Cultures (2 mL) inoculated from single colonies were grown overnight in LB medium with orbital shaking (200 rpm) at 37 °C. The overnight culture was plated (20 µL, OD600 = 0.5) to homogenously cover the detection areas of the coated dish.

**Scheme 1:**
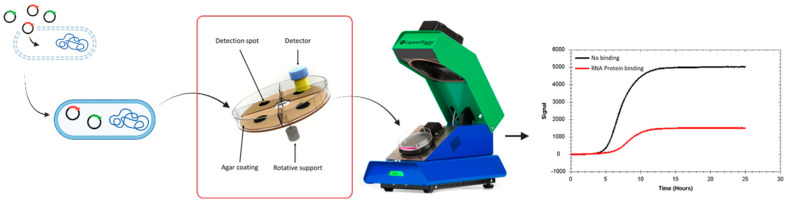
LigandTracer schematic setup for detecting RNA–protein interactions in living bacteria. Created in BioRender. Perez, G. (2025) https://BioRender.com/c32f073 (accessed on 22 January 2025).

*LigandTracer.* After seeding bacteria, MultiDishes were placed on the rotating support in LigandTracer Green (1-04-002, Ridgeview Instruments) (Scheme 1). To detect fluorescence from sfGFP, the LigandTracer was equipped with a BlueGreen (488–535 nm) detector, and the assay was designed to detect fluorescence coming from the four different detection spots for 15 s per spot, resulting in a data collection frequency of 1 min^−1^.

*Data evaluation*. The Gompertz model, a mathematical model used to fit sigmoidal growth curves, was used to fit the obtained signal vs. time curves. The used equation generates three parameters: the maximum signal at the curve asymptote (A), the growth-rate coefficient (k), and the time of inflection (T) [43].

The model has been used to fit biological growth data since 1926 and has been applied to microbial and tumour growth. Here, we used a type I, three-parameter model, where two of the parameters affect the curve shape (A and k) and a third one marks the shift in the curve in time without affecting its shape (T) [43].

The reduction in the maximum signal at the curve asymptote (A) was the main parameter used to evaluate the effect of protein–RNA interactions. All conditions were evaluated in replicate. In some cases, bacterial growth was severely delayed. To assure data quality and robustness, curves that had two-fold higher inflection than the average of other replicates were excluded.

## 3. Results

### 3.1. Real Time Protein Expression Assay (RT-PEA) in Living Bacteria

**Figure 1:**
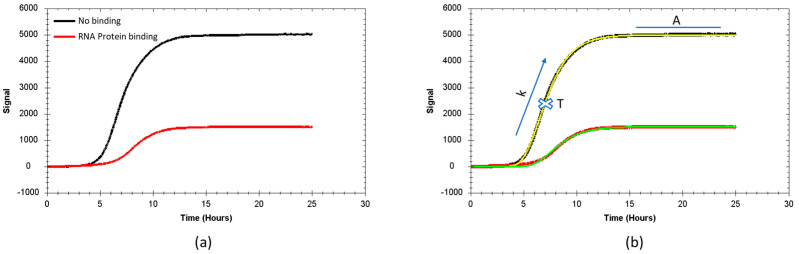
Representative signals as a function of time, obtained from the reporter system and a schematic representation of the theoretical traces obtained from the fitting. (**a**) In black, the fluorescence signal over time corresponds to bacteria where MSI1 expression has not been induced. In red, the fluorescence signal over time corresponds to bacteria where MSI1 expression has been induced. (**b**) Experimental results (black and red lines) with their corresponding fit (yellow and green lines) after applying the Gompertz model. The obtained parameters are superimposed in the graph: maximum fluorescence signal (A), maximum sfGFP expression rate (k × A) at the inflection point, and the inflection point (T) itself.

To enable the evaluation of regulatory effects of RNA–protein interactions in bacteria cultured on solid media, a new time-resolved method based on a fluorescent reporter system was developed. Fluorescent signals, generated by bacteria engineered as described previously [42], were monitored as function of time using a LigandTracer device. To validate the reporter system, bacteria were grown in the presence and absence of IPTG to induce MSI1 expression or not. The 1 mM IPTG concentration was 10-fold higher than the concentration required to obtain half of the maximal expression for the same reporter system when bacteria are grown in suspension [42]. In the same article, it was observed that IPTG-induced expression did not noticeably affect bacterial growth rates. To ensure that the number of cells was similar, the same volume of cells at an OD600 of 0.6 was seeded and uniformly spread over the agar in the detection area, and bacteria were grown until reaching a plateau phase or until enough curvature was generated to predict the final plateau signal. The dish was placed in the LigandTracer, and fluorescent signals at 535 nm, as emitted by sfGFP, were followed over time. The obtained curves were then subtracted from the signal of the agar without cells, obtaining background-corrected curves. The typical curve displayed a sigmoidal shape, with a lag phase of approximately five hours until bacteria started to produce the fluorescence protein sfGFP, first in a linear fashion that slows down after a few hours and finally reaches a plateau (Figure 1). The shape of the sfGFP signal over time resembles the first three phases of a traditional bacterial growth curve with a lag phase, an exponential phase with rapid cell growth, and a stationary phase. In a standard assay, fluorescence signals were monitored for more than 24h and the plateau signals were often stable for more than ten hours without observing a reduction in the signals. For example, the sfGFP curves shown in Figure 1 have a signal change from the beginning of the stationary phase after 15 h to the end of the measurement at 28 h of less than 1%. This implies not only that cell viability is maintained for a long time during the stationary phase, but also that photobleaching and/or phototoxicity is neglectable. Bacteria death, the fourth phase in a bacterial growth curve described as an acute signal descent, was not observed in any of the performed experiments, even when assay duration was extended to 48 h.

As the sfGFP signal as function of time resembles a bacterial growth curve, a well-known and widely used growth model was used to fit the sfGFP signal over time curves to extract three parameters: the maximum signal at the asymptote (A), the inflection time (T) (time in hours where signal equals e^−1^ of A), and the maximum relative growth rate (k), i.e., the growth rate at the inflection point relative to the maximum signal.

Protein binding to the primary RNA sequence was used to evaluate the performance of the developed method. This sequence is based on a previously reported screening study and contains the consensus recognition sequences GUUAGU and AUUUAGU [44]. The acquired curves (n = 8) showed a clear downregulation of sfGFP expression in the presence of IPTG-induced MSI1 expression, compared with the non-induced populations with a reduction in the plateau signal level of 58% (CV 27%). The average relative sfGFP growth rate, k, for the non-induced bacteria was 0.59 signal units·h^−1^ (CV 9%) while the sfGFP growth rate for the induced populations was slightly lower, with 0.50 signal units·h^−1^ (CV 19%). The time required to reach the inflection point from the start of the assay was 7.8 h (CV 7%) for the non-induced population, while this value was 10.4 h (CV 14%) for the IPTG-treated population.

In general, the expression rate was reduced and the time to reach the inflection point was longer when MSI1 expression was induced. Moreover, the expression rate was typically lower for assays in which the inflection point was reached later. It is thus possible that the additional burden on the cells when inducing MSI1 expression increases the lag phase and reduces the expression rate. The variation in k- and T-values, however, is not correlated to the variation in the overall reduction in the sfGFP signal upon MS1 expression. Therefore, further evaluation of other factors that influence MSI1-based regulation of sfGFP expression were performed based on the resulting relative reduction in the plateau signal level.

To discard the possibility that fluorescence signals could be affected by the experimental design, experiments with different IPTG concentrations were performed (1 mM, 0.75 mM, 0.5 mM and 0.25 mM), confirming that 1 mM IPTG produced a higher fold change (Figure S1). To check if another endogenous protein could interact with the system, bacteria were transformed with just the reporter plasmid, and fluorescence was monitored upon the addition of 1 mM IPTG in mutants M5 and M6 (Figure S2), without observing variations in the fluorescence fold changes. The inflection time was used as an indicator of data quality, considering that a large deviation between the non-induced and induced populations could indicate an affection of protein expression or cell growth. Therefore, a threshold of two-fold deviation between the inflection time of the induced and non-induced populations was established. This margin takes into account the fact that the extra burden posed by the expression of MSI1 can produce a delay in the inflection time point while, at the same time, excluding data where fluorescence expression is strongly delayed by external conditions. This threshold also ensures that the selected curves display sufficient curvature, which is a critical parameter for the Gompertz model to reliably fit the data.

### 3.2. Characterization of MSI1 Binding to RNA

**Figure 2:**
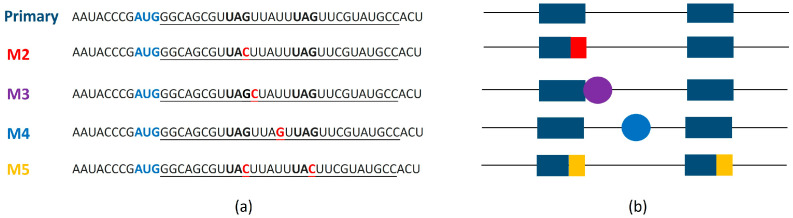
RNA sequences used in the reporter system. (**a**) Sequence of the mutants, with mutated nucleotides highlighted in red and the three interacting nucleotides of the core motif depicted in bold. The AUG motif depicted in blue is the starting codon. (**b**) Schematic representation of the mutations (each dark blue rectangle depicts the core motif for RNA binding).

Mutations in the core UAG motif have been related to a reduction in the binding affinity and are therefore expected to also affect the translational repression of sfGFP in the engineered system. Different RNA strands were designed to evaluate the influence of RNA-binding affinity on the sfGFP signal (Figure 2). One of the selected strands (Mutant 2, M2) harbors a single-nucleotide mutation affecting one of the binding motifs. As in silico studies predicted, residues close to the UAG core motif can affect MSI1 binding [45] mutants 3 and 4 where selected that contain a single mutation outside the binding motif. Mutant 3 (M3) has a U-to-C mutation adjacent to one of the binding motifs, while mutant 4 (M4) was mutated in the linker between both binding motifs. Mutant 5 (M5) has a G-to-C mutation in both binding motifs. (Figure 2)

**Figure 3:**
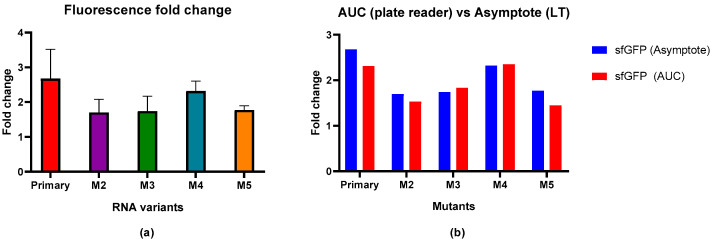
Fluorescence signal changes upon MSI1 expression in solid media and comparison with results from bacteria in suspension. (**a**) Fluorescence fold change upon MSI1 binding to different RNA mutants in bacteria in solid media. Results are shown as the average of the replicates with error bars corresponding to the standard deviation. (**b**) Comparison of fluorescence fold change between current results performed with the reporter circuit in solid media (max signal fold change) and results from experiments with bacteria in suspension (AUC fold change) (Dolcemascolo et al., 2024) [42].

Having the primary RNA sequence before the sequence coding for sfGFP resulted in a 2.68-fold reduction in the max fluorescence signal compared to the non-induced population as shown in Figure 3a. Mutants M2 (1.70-fold), M3 (1.74-fold), and M5 (1.77-fold) showed similar reductions in the fluorescence signal, that were clearly lower than the wild-type (primary)-induced reduction, indicating the weaker effect of MSI1 regulation on sfGFP expression. Mutant 4, with a mutation in the sequence between both RNA-binding motifs, has a 2.32-fold reduction in the fluorescence signal, closer to the wild-type value. Although the individual signal reductions observed for the mutants were not statistically significant, based on a one-way ANOVA statistical analysis, there is a clear trend that mutations in or adjacent to the UAG core motif led to a reduced inhibition of sfGFP expression.

To validate the new method and check its reproducibility, the obtained results were compared with the results obtained by using bacteria grown in suspension and analyzed using a plate reader (Figure 3b). As observed from the resulting graph, there were no significant differences between the results obtained using these methods, with similar and consistent fold values for each interaction.

### 3.3. Correlation Between Fluorescence Reduction and Affinity

The binding affinity (K_D_) of purified human MS1 to the different RNA strands was previously characterized using SwitchSense technology and correlated to the reduction in fluorescent signals for suspension bacteria by plotting the fold change against the equilibrium binding constant (1/K_D_) resulting in a linear relation with r^2^ = 0.75 [42]. When plotting the currently obtained fold changes against the equilibrium binding constant, linear regression resulted in an even higher linearity (r^2^ = 0.89).

### 3.4. Oleic Acid Allosteric Modulation of MSI1 Binding to RNA

**Figure 4:**
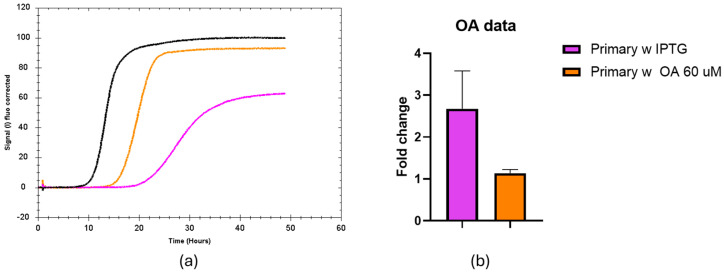
Effect of oleic acid on the fluorescent signal over time from the MSI1-RNA reporter system. (**a**) Representative trace of the fluorescence signals corresponding to black bacteria seeded in LB agar without IPTG-induced MSI1 expression, orange bacteria seeded in 60 µM oleic acid in presence of IPTG and pink bacteria seeded in LB agar with IPTG. (**b**) Fluorescence fold change in the primary strand upon induction of MSI1 expression with IPTG, either combined (orange) or not (pink) with oleic acid treatment. A *t*-test was applied to compare both conditions, showing a meaningful difference between the treated and untreated groups with a *p*-value of 0.0179.

MSI1 binding to RNA can be allosterically inhibited by mono-unsaturated fatty acids such as oleic acid. To assess if the current setup can monitor this regulation, 15 to 60 µM oleic acid was included in the agar plate. We found a stronger effect when using 60 µM (Figure 4). We quantified that upon the addition of oleic acid and IPTG, the fluorescence signal during the stationary phase is similar to the situation where MS1 expression was not induced, and the fold change was only 1.14. Thus, oleic acid strongly counteracts the effect of IPTG-induced expression of MSI1 on sfGFP production.

## 4. Discussion

Reporter gene assays represent one of the basic tools in molecular biology, which can be used in multiple organisms, from bacteria to animals, to study complex interactions and regulatory systems [46]. When using bacteria, reporter gene assays are generally performed with populations grown in liquid media. Cell suspension in a liquid medium has clear advantages, such as fast growth and analytical instrumentation, that allow for high-throughput analysis. However, not all bacteria reporter assays are suitable to be performed in suspension, as transformed cells carry the burden of extra genetic material that has to be replicated, transcribed, and translated [47]. This extra burden, in combination with the presence of external inducers at high concentrations, can influence the outcome of the assay, affecting bacteria growth and fluorescence signal detection. Bacteria that can be better characterized using solid media are biofilm-forming species, such as *Pseudomonas aeruginosa* [48], and endospore-forming bacteria, such as some species of the genus *Bacillus* [49].

The combination of seeding agar plates and LigandTracer technology allows for a relatively high data collection frequency while offering an optimum environment for bacteria to grow. Conditions which are beneficial for bacterial growth produce experimental results that often reach a long-lasting stationary phase and enough curvature to calculate the maximum signal with confidence. In liquid media, equilibrium is not always reached. This is especially true in slowly growing bacterial strains such as *Mycobacterium smegmatis* [50]. As cells are seeded in similar quantities and grow until reaching confluence and a stable stationary phase, the reported fold changes, based on relative sfGFP signals, do not require normalization on the number of bacteria in the detection area. The high cell viability also enabled to perform assays with oleic acid (a small molecule that binds MSI1 and prevents RNA recognition) that were difficult to perform for bacteria in suspension [42]. LigandTracer technology is designed to monitor the binding processes of fluorescent compounds for many hours and is therefore equipped with a low-power light source to minimize photobleaching. This appears to be an ideal platform to characterize regulatory systems in bacteria, allowing for the study of dynamic behaviors in highly complex environments such as biofilms [51].

The time-course of sfGFP signals was analyzed by fitting the Gompertz model to the experimental data. This empirical model has relatively few parameters compared to ab initio kinetic models [42]. Absolute fluorescence signals closely resembled growth curves without indication that the reporter assay was regulated in a complex manner. While more advanced models potentially enable the extraction of more details, limiting the number of parameters to be fitted reduces the risk of overfitting and enhances the general applicability with focus on the relating expression regulation to protein–RNA interactions, even though sfGFP expression level in a bacterial cell over time depends on a cascade of processes such as transcription, translation, folding, and degradation [52].

A clear change in the measured signal upon inducing MSI1 expression with IPTG (comparison of stationary phases) was observed, and this change correlated well with the expected binding efficiency of MSI1 to RNA. This confirms that the translation repressing function of MSI1 in mammals can be transferred to bacteria to engineer post-transcriptional control circuits. The results obtained on the fluorescence fold change and the expected affinity aligned well with previously published data on the same system [42], as well as with the expected behavior related to the binding, suggesting the utility of the new method to study RNA–protein interactions in a physiological environment. Previously published studies on MSI1-RNA binding [17] postulated that bivalent binding is readily established, leading to an increase affinity due to avidity. For this bivalent binding to occur, the presence of two free RNA-binding motifs is required. This is the case with the primary and M4 RNA strands, which are indeed the ones causing a higher fold change in the fluorescence, therefore aligning with the expected behavior based on in vitro experiments. The other mutants, M2 (with one binding motif mutated), M3 (with a mutation adjacent to the binding motif) and M5 (with both binding motifs mutated) showed similar fold change reductions in the fluorescence that were lower than those observed for the primary and M4 RNA strands. This similar behavior was confirmed by the RRMscorer software (v.2024.12.0), which gave similar values (representing binding likelihood) for the three mutants against both RRM1 and RRM2 (Table S1). The lack of bivalent binding patterns for mutants M2, M3, and M5 was confirmed by Dolcemascolo et al., where it was shown that these mutants interacted in a classical one-to-one binding pattern. One-to-one interaction of single MSI1 RRMs to RNA showed a high affinity, but with a fast dissociation, explaining that a fold change variation is still observed during dissociation when both UAG core motifs are unchanged. These different interaction modes are visible in the interaction patterns shown by Dolcemascolo et al., where interaction patterns of MSI with the wild-type and M4 showed biphasic dissociation.

The relationship between fluorescence fold-change and affinity showed a better linearity when compared with the findings of Dolcemascolo et al. However, it should be noted that the good linear relationship is largely due to the existence of two distinct affinity populations, which are coincidental with the kinetic binding mode described. MSI1 binding M2, M3 and M5 showed a one-to-one interaction pattern, and their equilibrium binding constants cluster together, while another cluster was formed by primary and M4, which displayed a biphasic dissociation pattern.

The binding of MSI1 to RNA is allosterically regulated by the binding of monounsaturated fatty acids to the RRM1 domain. It has been proposed that binding of oleic acid to the RRM1 of MSI1 inhibits RNA binding [40]. As oleic acid only affects one of the two RRMs of Musashi, this would disrupt the bivalent binding of the protein and turn it into a one-to-one interaction, which would produce a lower fold change in the fluorescence signal. The characterization of the allosteric interaction in the bacterial population would add a new layer of information on the modulation of protein–RNA binding that could be used to the search of compounds with potential to affect those interactions. The results of the experiments performed in the presence of oleic acid showed an almost complete reduction in the effect that MSI-RNA binding has on sfGFP expression. This reduction in sfGFP expression is stronger than expected, as theoretically only the RRM1 would be affected and MSI1 could still bind in a one-to-one fashion with its second RRM2 domain. Therefore, a fold change would be similar to the RNA mutants with a single RNA motif available. Previous attempts to study allosteric modulation in suspension bacteria failed in a plate reader assay due to the turbidity of the medium but could be characterized in single bacteria through flow cytometry [42]. This method lacks time resolution, while the solid support media allows for the use of complex solid media that, together with the high time-resolution of the LigandTracer and the possibility of running longer times, makes the new method suitable for monitoring this modulation.

Although this system is well-suited to characterize RBP-RNA interactions in a physiological setting, the complexity of RBP-RNA interactions requires a careful design of the reporter circuit. RBPs are known to bind to short RNA motifs that are common across different mRNA, while specificity is enhanced by avidity, i.e., a multivalent interaction [17].

A multivalent interaction, however, also adds a layer of complexity to the design of the reported system, as self-competition can affect the outcome. Moreover, the avidity effect can depend on the distance between RNA motifs [53], the presence of RNA secondary structures [54], or the presence of other biomolecules that affect the complex [41]. These conditions should be assessed when designing the RNA sequence to be used in the reporter assay, for example, through computational simulations or based on results from in vitro experiments on the interaction.

The overall approach, however, is suitable for the current reporter assays with the purpose of investigating how translation is regulated by RBP-RNA interactions in a physiological environment and adds a new method to the existing toolbox of molecular biology techniques used in the field.

## Data Availability

Data are contained within the article and supplementary materials.

## Supplementary Materials

The following supporting information can be downloaded at: https://www.mdpi.com/article/10.3390/bios15030175/s1, Figure S1: Fluorescence fold change upon different IPTG concentrations (0.25, 0.5, 0.75 and 1 mM) tested with Musashi-1 and the primary RNA sequence; Figure S2: Fluorescence fold change of Mutants M4 and M5 transformed only with the reporter plasmid and treated with IPTG, compared with bacteria transformed with both plasmids and non-treated with IPTG; Table S1: RRMScorer software was used to predict the binding likelihood of MSI1 to the different RNA motifs.

## Abbreviations

The following abbreviations are used in this manuscript:

CLIP	Crosslinking and immunoprecipitation
EMSA	Electrophoretic mobility shift assay
IPTG	Isopropyl β-D-1-thiogalactopyranosi
LB	Lysogeny broth
MSI1	Musashi-1
NMR	Nuclear magnetic resonance
RBP	RNA-binding protein
RNA	Ribonucleic acid
RRM	RNA recognition motif
RT-CBA	Real-Time Cell Binding Assay
RT-PEA	Real-Time Protein Expression Assay
sfGFP	Superfolder GFP
SPR	Surface plasmon resonance
