# Supplementary material for: Regulatory Effects of RNA–Protein Interactions Revealed by Reporter Assays of Bacteria Grown on Solid Media
Source: Biosensors (Basel). 2025 Mar 8;15(3):175. doi: 10.3390/bios15030175 (PMC11940492; doi:10.3390/bios15030175)
Supplement: Supplementary file 1 [file biosensors-15-00175-s001.zip › biosensors-3468469-supplementary.pdf]

Supplementary Information

Fluorescence fold-change upon different IPTG concentrations (0.25, 0.5, 0.75 and 1 mM) tested with Musashi-1 and the primary RNA sequence.

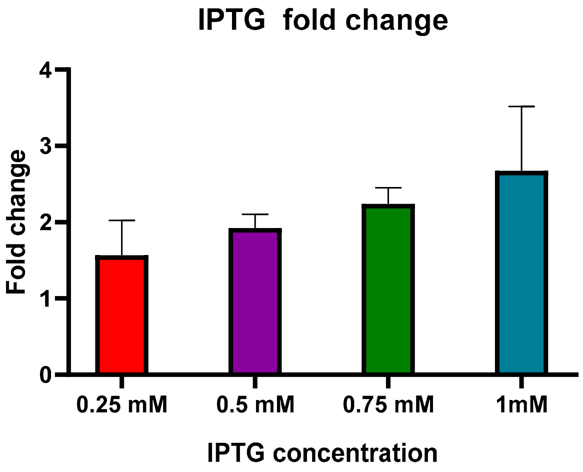

Figure S1: Fluorescence fold change at different IPTG concentrations

Fluorescence fold-change of Mutants M4 and M5 transformed only with the reporter plasmid and treated with IPTG, compared with bacteria transformed with both plasmids and non-treated with IPTG.

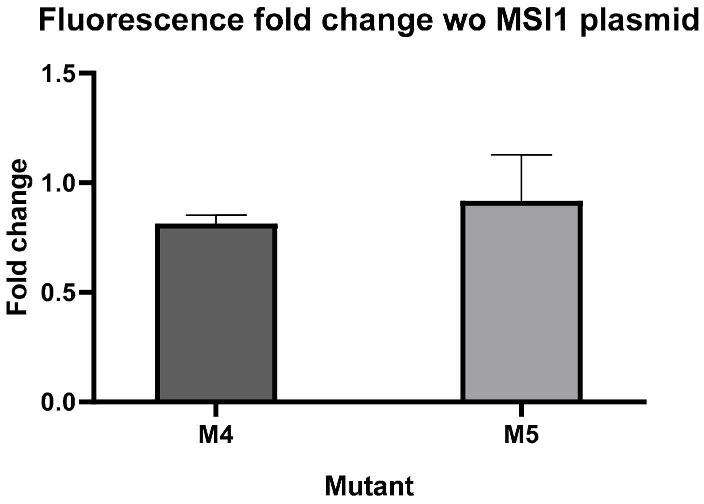

Figure S2: Fluorescence fold-change of Mutants M4 and M5 transformed only with the sfGFP encoding plasmids and treated with 1 mM IPTG, compared with bacteria transformed with both plasmids and non treated.

RRMScorer software was used to predict binding likelihood of MSI1 to the different RNA motifs (Roca-Martínez et al., 2023).

Table S1. Values obtained from RRMscorer software regarding RRM1 and RRM2 interactions with the sequences contained in the different RNA strands.

RNA strand	Sequence	RRM-1 Scorer	RRM-2 Scorer	Average Scorer
Primary	UUAGU	-0,248	-0,116	-0,182
M2	UUACU	-0,476	-0,431	-0,4535
M3	UUAGC	-0,458	-0,409	-0,4335
M4	UUAGU	-0,248	-0,116	-0,182
M5	UUACU	-0,476	-0,431	-0,4535
